# Proactive Supply Chain Performance Management with Predictive Analytics

**DOI:** 10.1155/2014/528917

**Published:** 2014-10-15

**Authors:** Nenad Stefanovic

**Affiliations:** Institute of Mathematics and Informatics, Faculty of Science, University of Kragujevac, Radoja Domanovica 12, 34000 Kragujevac, Serbia

## Abstract

Today's business climate requires supply chains to be proactive rather than reactive, which demands a new approach that incorporates data mining predictive analytics. This paper introduces a predictive supply chain performance management model which combines process modelling, performance measurement, data mining models, and web portal technologies into a unique model. It presents the supply chain modelling approach based on the specialized metamodel which allows modelling of any supply chain configuration and at different level of details. The paper also presents the supply chain semantic business intelligence (BI) model which encapsulates data sources and business rules and includes the data warehouse model with specific supply chain dimensions, measures, and KPIs (key performance indicators). Next, the paper describes two generic approaches for designing the KPI predictive data mining models based on the BI semantic model. KPI predictive models were trained and tested with a real-world data set. Finally, a specialized analytical web portal which offers collaborative performance monitoring and decision making is presented. The results show that these models give very accurate KPI projections and provide valuable insights into newly emerging trends, opportunities, and problems. This should lead to more intelligent, predictive, and responsive supply chains capable of adapting to future business environment.

## 1. Introduction

Today, supply chains are very complex business networks that need to be managed collaboratively and optimized globally. Additionally, global business landscape is constantly and rapidly changing. Uncertainty, growing competition, shorter cycle times, more demanding customers, and pressure to cut costs are just a few characteristics of the 21st century business environment. It has become critical to measure, track, and manage the performance of supply chain processes. Performance management relates to application of processes, methods, metrics, and technologies in order to create a consistent relationship between supply chain strategy, planning, implementation, and controlling.

Supply chain requires that member companies have the means to assess the performance of the overall supply chain to meet the requirements of the end customer. In addition, it is necessary to be able to assess the relative contribution of individual member companies within the supply chain. This requires a performance measurement system that can not only operate at several different levels but can also link or integrate the efforts of these different levels to meet the objectives of the supply chain [1]. In order to accomplish this requirement, the performance measurement process will need to provide methods and tools for measuring, monitoring, and managing supply chain processes. Supply chain management (SCM) has received a remarkable attention from both academia and industry since the last decade; however, there is still lack of integration between SCM systems and performance management systems. The huge majority of performance measurement models and frameworks have focused on single organizations or cover specific type of performance such as financial. There are several performance measurement approaches specifically designed for the supply chain management domain [2]. Companies have to measure performance at strategic, tactical, and operational levels with metrics dealing with sourcing, making, delivering, and customer services [3].

When companies use standardized metrics they can join benchmarking databases and use benchmarking services to compare with best in class companies and perform gap analysis. This analysis identifies weak points in the supply chain that require some improvement through process redesign or reengineering. Furthermore, standardized metrics facilitate collaboration and integration within the supply chain and with 3rd party logistic providers and outsourcing companies. Such collaboration models are based on a clear definition of what is expected from the partners and service providers in terms of process performance. Supply chain collaboration improves competitive advantage and enables supply chain partners to achieve synergies and create superior services.

In order to achieve required supply chain agility and adaptivity, it is necessary to use intelligent technologies and tools which enable monitoring and evaluation of supply chain performance. To be competitive, companies have to utilize business intelligence (BI) technologies and tools in order to better manage their businesses and anticipate the future. Combination of business intelligence and performance management systems can improve supply chain efficiency and accountability and reduce costs with optimized decision-making process based on monitoring of the key performance indicators. In addition, these systems should enable more predictable performance management by providing actionable information to the right decision makers. The resulting increased demand for business intelligence means that companies should focus on the goal of providing all stakeholders with the right information at the right time with the right tools. Achieving this objective requires the use of BI solutions and applications for tracking, analyzing, modelling, forecasting, and delivering information in support of performance management and decision-making processes [4].

Performance management (PM) complements BI and links people, strategies, processes, and technology. The PM system can be a platform for the improvement of supply chain operations. It usually provides information about what happened, why something happened, and appropriate courses of actions. The main goal of supply chain PM systems is business process optimization through monitoring and analysis of key performance indicators (KPIs). These performance measures enable supply chain companies to align processes and activities with strategic objectives.

KPIs are often used in BI systems to measure the progress of various metrics against business goals. They have become very popular for BI analysis because they provide a quick and visual insight into measurable objectives. KPIs are customizable business metrics that present an organization's status and trends toward achieving predefined goals in clear and user friendly format. After a supply chain or member company defines its strategy and objectives, KPIs can be defined to measure its progress toward those objectives. KPIs are becoming essential elements of supply chain performance management software, balanced scorecards, and analytical dashboards.

Although more and more companies use KPIs for measuring business performance, these key performance indicators are typically internal, financial, and functional. Financial accounting measures are certainly important in assessing whether or not operational changes are improving the financial health of an enterprise but are insufficient to measure supply chain performance for the following reasons [5].

- The measures tend to be historically oriented and not focused on providing a forward-looking perspective.
- The measures do not relate to important strategic, nonfinancial performance.
- The measures are not directly tied to operational effectiveness and efficiency.
- Most performance measurement systems are functionally focused.

Supply chain performance measurement requires specific metrics which are global, process based, multitiered, and comprehensive.

The problem with most of existing PM systems is twofold. The first one is related to data. Supply chains usually hold a vast amount of distributed and heterogeneous data. Integration of this isolated and often incompatible data is a big challenge. To be able to make better decisions based on facts, organizations need to get this factual information typically from several information systems, integrate this data in a useful way, and present users with reports and analysis that will help them to understand the past and the present organizational performance.

Secondly, KPIs are traditionally retrospective, for example, showing last month's stock level compared to the stock target. However, with insights made possible through data mining, organizations can build predictive KPIs that forecast future performance against targets, giving the business an opportunity to detect and resolve potential problems proactively.

The next step in promoting supply chain agility and operational efficiency is to make the leap from retrospective analysis of historical KPIs to proactive actions based on predictive analysis of supply chain performance data and to embed intelligent, fact-based decision-making into business processes. The key to accomplishing this is to use powerful data mining algorithms to analyze data sets, compare new data to historical facts and behaviors, identify classifications and relationships between business entities and attributes, and deliver accurate predictive insights to all systems and users who make business decisions [6].

The remainder of this paper is organized as follows. It starts with literature review related to supply chain performance management and predictive analytics. Then, a unified approach to supply chain performance management which integrates supply chain process model, online analytical processing (OLAP), KPIs, data mining predictive analysis, and web portals is presented.

## 2. Background Research

Performance management represents a cohesive element that unites different business improvement initiatives, directs strategy formulation, and plays the key role in strategy implementation and monitoring. PM is of great importance for managing supply chains. Timely and accurate evaluation of the entire supply chain and individual companies is prerequisite for successful functioning. Performance management should be a part of any proper supply chain strategy, planning, and reporting process.

There has been lot of research efforts during the last decade that deal with various aspects of supply chain performance management. Shepherd and Gunter provide taxonomy of supply chain performance measurement systems and metrics with critical review of the contemporary literature [7]. The study shows that despite substantial advances in the literature in the last decade, there are still important topics related to supply chain performance management that did not receive adequate attention, such as process modeling, data integration, software support, and forecasting. Estampe et al. provide analysis of various supply chain performance models by stressing their specific characteristics and applicability in different contexts, so that decision-makers can evaluate and apply models and metrics that best suit their needs [8]. Arzu Akyuz and Erman Erkan also provide a critical literature review on supply chain performance measurement. The results show that performance measurement in the new era is still an open area of research [9]. This particularly refers to framework development, collaboration, agility, flexibility, and IT support systems. Gopal and Thakkar give a comprehensive review of supply chain performance measurement systems [10]. The analysis shows that, in spite of considerable evidence from the literature, there is a large scope for research to address critical issues in supply chain performance measurement, including metrics, benchmarking, integration, business intelligence, and collaborative decision making.

The goal of supply chain performance management is business process optimization through monitoring and analysis of key performance indicators. By measuring and monitoring metrics against predefined goals companies can provide added value to large volumes of data generated over time. This type of analysis allows companies to track various metrics at different organization levels and to take timely actions.

This has elevated the importance of key performance indicators and their ability to measure, predict, and manage the business health of a supply chain in real (or near real) time. KPIs have morphed from static siloed measures to dynamic real-time enterprise metrics. Key performance indicators are normally backward-looking because they are based on historical information and often do not help in forecasting future events or performance [11]. Predictive metrics would make it possible to predict future problems in supply chain operations and to enable proactive evaluation and improvement actions in advance. Statistical modelling and data mining under the guise of predictive analytics have become critical building blocks in setting the new KPI standard for leading indicators [12].

However, most of the existing performance measurement systems are mostly based on financial indicators (i.e., costs). They are also internally focused, incompatible, and historical. In today's business environment this performance measurement approach is no longer adequate. New business climate requires novel and innovative performance management systems which have the following characteristics [13]:

- directly related to overall strategy,
- comprehensive (takes into account relevant variables),
- use also non-financial KPIs,
- adaptable to specific supply chain configuration and member companies,
- optimal number of KPIs,
- simple and easy to use,
- timely and actual,
- accurate and consistent,
- foster improvement, not just monitoring,
- represent information at different supply chain levels,
- provide analytical tools that offer multidimensional reporting,
- enable a proactive management, rather than reactive,
- provide insights into newly emerging trends, opportunities and problems,
- deliver information to right people at any time and on any device.

In response to these challenges for measuring supply chain performance, a variety of measurement approaches have been developed, including the following: the balanced scorecard, the supply chain council's SCOR model, the logistics scoreboard, activity-based costing (ABC), and economic value analysis (EVA) [5]. Lambert and Pohlen introduced a framework for developing metrics that measure the performance of key supply chain processes, identify how each firm affects overall supply chain performance, and can be translated into shareholder value [14]. Cai et al. propose a framework for analyzing supply chain KPIs accomplishment, so that management strategy can be attuned by interpreting the analysis results [15]. This framework aligns performance at each link (supplier-customer pair) within the supply chain. Gunasekaran et al. developed a framework for supply chain performance measures and metrics considering the four major supply chain processes: plan, source, make, and deliver [16]. Metrics were classified at strategic, tactical, and operational levels in order to clarify management authority and responsibility for performance.

In an attempt to overcome some limitations of traditional PM systems, many companies have initiated balanced scorecard (BSC) projects. Based on the methodology of Robert Kaplan and David Norton, these companies created a balanced set of metrics representing financials, customers, internal business processes, and innovation [17]. The goal was to enable better decision making by providing managers with a broader perspective of both tangible and intangible assets. However, the supply chain has specific processes and metrics which require special types of balanced scorecards. Bhagwat and Sharma proposed a balanced scorecard for supply chain management that measures and evaluates day-to-day business operations from the four BSC perspectives and with specific SCM metrics [18].

**Figure 1:**
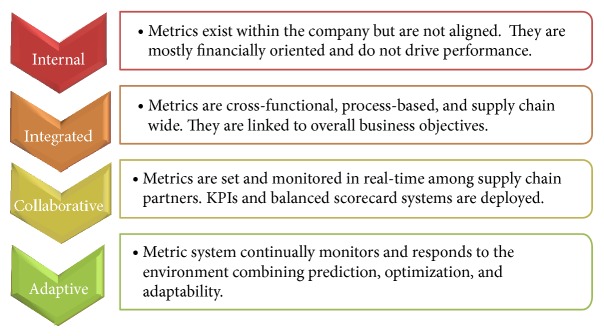
Supply chain performance measurement maturity model.

Only a few leading-edge companies are currently using true extended PM systems (either in-house developed or implemented PM software applications) that not only measure the performance of their enterprise but also measure that of their supply chain wide activities. Most companies are still in the* internal* or* integrated* stage of the maturity model (as shown in Figure 1) where they focus on the performance of their own enterprise and measure their supply chain performance with financially oriented metrics [19].

One of the prerequisites for effective supply chain performance measurement is the initiative geared toward standardization of supply chain processes and metrics. Standardized models facilitate application integration and collaboration, enable benchmarking for performance comparison, and provide best practices for process improvement and gaining a competitive advantage. By standardizing supply chain processes and metrics for managing such processes, companies are able to not only compare their results against others, but to also gain visibility of operations over a supply chain that may cross corporate borders. Partners in a supply chain can communicate more unambiguously and can collaboratively measure, manage, and control their processes [20].

SCOR (supply chain operation reference) model represents a universal approach to supply chain management that can be applied in diverse business domains [21]. SCOR combines business process engineering, benchmarking, and best practices into a single framework. This standardization movement facilitates the deliverance of business content for the supply chain KPI and predictive analysis model. Kocaoğlu et al. propose a SCOR based approach for measuring supply chain performance [22]. It uses the analytic hierarchy process (AHP) and technique for order preference by similarity to ideal solution (TOPSIS) together for the linking of strategic objectives to operations.

Although the SCOR model provides a layered metric system and thereby a business context for the KPI system, it is only the first step toward the pervasive supply chain performance measurement. In order to implement such a complex PM system as found in supply chains, member companies need to extract, transform, and load all the relevant data into a unique and integrated data source.

Supply chain integration is critical to both operational and business performance. In order to achieve the full effect of supply chain integration, companies need to use collaborative and intelligent web information systems to actively manage process uncertainty. Most of the existing information systems that support supply chain management are repositories for large amounts of transactional data. These systems are said to be data rich but information poor. The tremendous amount of data that is collected and stored in large, distributed database systems has far exceeded the human ability of comprehension without analytic tools. Analysts estimate that 80% of the data in a transactional database that supports supply chain management is irrelevant to decision making and that data aggregations and other analyses are needed to transform the other 20% into useful information [23].

As a result of the huge amount of data generated within supply chains, new tools and methods should be developed which are capable of storing, managing, and analyzing the data, as well as of monitoring global supply chain performance. Chae and Olson propose an analytical framework for supply chain management, which is composed of three IT-enabled capabilities: data management, analytics, and performance management [24].

Increasingly, transactional supply chain data is processed and stored in enterprise resource planning systems, and complementary data warehouses are developed to support performance management and decision-making processes [25]. Some organizations have developed data warehouses and integrated data-mining methods that complement their supply chain management operations [26, 27].

Investment in business intelligence software is necessary if the organizations want to manage their supply chain more effectively. The studies show that main drivers for BI initiatives include complexity of operations, huge amounts of data, requirements for cost reduction, increased revenue, and efficiency improvement, as well as high exposure to risks [28]. Data warehousing and online analytical processing technologies combined with tools for data mining and knowledge discovery have allowed the creation of systems to support organizational decision making [29].

Inventory management is probably the key supply chain process, and inventory costs represent a large portion of the total supply chain costs. Incorporating predictive analytics in an inventory management process can lead to many benefits such as cost reduction, higher customer service level, optimal reordering policy, enhanced productivity, shorter cash-to-cash cycle time, and ultimately increased profitability [30]. Different data mining techniques were used for solving supply chain and inventory management related problems. Dhond et al. used neural-network based techniques for inventory optimization in a medical distribution network which resulted in 50% lower stock levels [31]. Symeonidis et al. applied data mining technology in combination with an autonomous agent to forecast the price of the winning bid in a given order [32]. When it comes to inventory management, Stefanovic et al. used different data mining models clustering retail stores based on sales patterns and one/two weeks out-of-stock forecasting [33].

When data mining predictive analysis capabilities are closely integrated into every stage of the data life cycle, they incorporate intelligence into reporting, data integration, OLAP analysis, and business performance monitoring. This helps supply chains to increase business agility and creates a tangible competitive advantage.

As supply chain performance management systems mature, it is almost certain that predictive analytics will be an integral part of future PM and BI solutions. Predictive analytics leverage historical and current performance data in order to make predictions on future performance. For example, there could be a predictive analytical model that can make predictions about supply chain management costs, perfect order fulfillment, return on working capital, and so forth. The main difference between predictive analytics and classic BI is that predictive models contain an additional stage which utilizes certain data mining algorithms to predict future performance outcomes. This information can be used as a valuable resource for the decision-making process and refinement of strategic, tactical, and operational supply chain plans. One recent survey showed that 87 percent of respondents said that they think predictive analytics is important to the budgeting and planning process, but only 17 percent are employing technologies that include the capability [34]. Other studies show that the ROI of predictive mining applications is almost five times greater than that of nonpredictive applications using standard query, reporting, and analysis tools [35].

Although with great practical potential, there have not been many research projects related to predictive performance management. Seifert and Eschenbaecher introduced a predictive performance measurement approach as a planning tool for virtual organizations to anticipate the performance of a planned virtual team [36]. Derrouiche et al. proposed an integrated framework for supply chain performance evaluation based on data mining techniques [37]. It enables the development of a predictive collaborative performance evolution model and decision making which has forward-looking collaborative capabilities. Maleki and Cruz-Machado proposed a framework for developing data mining models based on Bayesian networks which account uncertainty and mutual dependency among supply chain performance measures [38].

Predictive KPI analytical models can identify critical KPIs and their likely impact on other indicators, both at the same level or higher-level KPIs. Besides typical predictive data mining models, other models can be created in order to further clarify prediction results, analyze key influencers, and perform scenario analysis such as goal seeking and what-if analysis. Thus, predictive PM systems will not only notify users about KPI risks, but will also provide additional information necessary for taking corrective actions.

On the other hand, most of the existing PM systems are not business user-friendly which makes them less attractive for wider implementations. Modern PM systems have to be web-based, interoperable, modular, and customizable.

Based on the analysis of the existing research results and also the methods and tools used in real-world supply chain operations, two main conclusions can be derived.

- Organizations show a strong interest in proactive supply chain performance management, but very few PM projects deal with predictive analytics.
- Most of existing research efforts, methods, and tools are targeted to specific supply chain PM aspects, without a comprehensive approach which would provide organizations with concrete models, methods, and tools for achieving business objectives.

What is needed is a unified supply chain performance management system to collect, integrate, and consolidate all relevant data and to use business intelligence tools like data warehousing and data mining, to discover hidden trends and patterns in large amounts of data, and finally to deliver derived knowledge to business users via web portals. In this context, a novel supply chain PM model and software solution that fulfills most of the previously stated requirements are proposed. In the subsequent sections, more information related to supply chain OLAP modelling, KPI design, data mining KPI prediction model, and PM web portal is presented.

## 3. Predictive Supply Chain Performance Management Model

Performance management of complex business networks such as supply chains requires a unified approach that comprises different management models, technologies, and tools. This section introduces an integrated supply chain PM model which incorporates the supply chain modelling method and business intelligence technologies such as data warehousing and data mining. It is based on the integrated supply chain intelligence model for collaborative planning, analysis, and monitoring [39].

**Figure 2:**
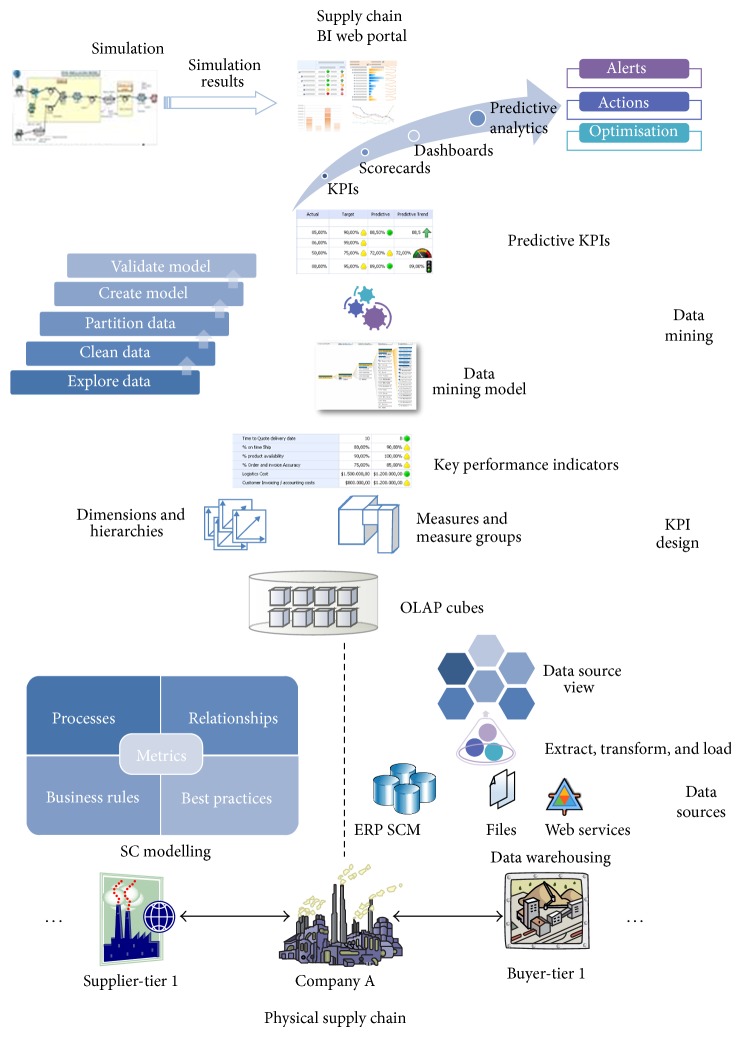
Predictive supply chain performance management model.

The main elements and the structure of the supply chain PM model are shown in Figure 2.

The basis of the model is the supply chain modelling method which enables modelling of supply chain processes, relationships, metrics, best practices, and other relevant elements. Output of this stage is a supply chain process model that serves as input for data warehouse design.

First, based on the process model, data from various sources is extracted, cleaned, and transformed in order to accommodate requirements for KPI design, multidimensional analysis, and data mining models. The following step is construction of OLAP cubes with proper dimensions and measures. OLAP schema is the basis for design of supply chain KPIs which measure the progress toward predefined goals. KPI typically provides a visual representation of metrics over time, rather than just displaying the numbers.

The next step which includes data mining is the key step toward predictive performance management. Here, historical performance (KPI) data is used for making predictions about future performance. This information is then delivered to decision making via a special BI web portal in the form of web reports, charts, scorecards, dashboards, or notifications. Alternatively, prediction information can be used in supply chain simulation models for analyzing different scenarios and risks [40]. Abukhousa et al. used simulation models as an analysis tool for predicting the effects of changes to existing healthcare supply chains and as a design tool to predict the performance of new systems under varying sets of input parameters or conditions [41].

The final step is taking appropriate actions to resolve problems and make adjustments to strategy and plans. These actions can be made based on more detailed reporting, data exploration, specific expert systems, simulation, and data mining models. One such intelligent software solution for integrated and interactive supply network design, evaluation, and improvement is developed [42]. It consists of three modules designed for knowledge-based supply network modelling, rule-based simulation executions, and intelligent assessment and improvement.

In the next subsections, the main elements of the proposed supply chain predictive PM model will be described.

### 3.1. Supply Chain Process Model

The starting point is the SCOR process model which provides a library of the supply chain specific set of processes, relationships, metrics, and best practices. The SCOR process model contains a standard name for each process element, a notation for the process element, a standard definition for the process element, performance attributes that are associated with the process element, metrics that are associated with the performance attributes, and best practices that are associated with the process.

All process metrics are an aspect of the performance attribute. Performance attributes for any given process are characterized as either customer-facing (reliability, responsiveness, and flexibility) or internal-facing (cost and assets) metrics.

Top level metrics are the calculations by which an implementing company can measure how successful they are in achieving their desired positioning within the competitive market space. Lower level calculations (levels 2 and 3 metrics) are generally associated with a narrower subset of processes. For example, delivery performance is calculated as the total number of products delivered on time and in full, based on a committing date. Additionally, even lower level metrics (diagnostics) are used to diagnose variations in performance against plan. For example, a company may wish to examine the correlation between the request date and committing date.

Each process from the process model has its related metrics, best practices, and inputs and outputs. All metrics follow the same template which consists of the following elements:

- name,
- definition,
- hierarchical metric structure,
- qualitative relationship description,
- quantitative relationship (optional, if calculable),
- calculation,
- data collection.

**Figure 3:**
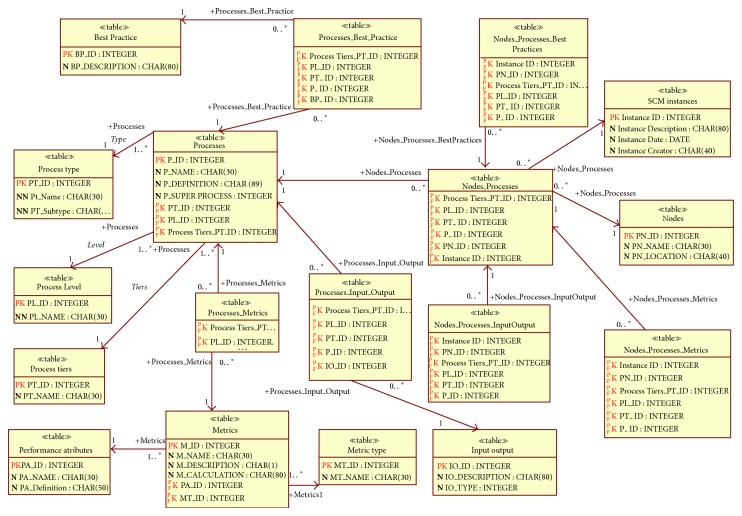
SCM metamodel.

Based on the SCOR process model, we have created the SCM metamodel (Figure 3), which enables the creation of any supply chain configuration and is the basis for further modeling [43]. Metamodel is normalized and contains all SCM elements such as processes, metrics, best practices, inputs, and outputs. It also incorporates business logic through relationships, cardinality, and constrains.

Metamodel is extended with additional entities to support supply network modelling. That way, processes, metrics, and best practices can be related to a specific node and tier in the supply network. With this metamodel, processes at different levels can be modelled, thus providing a more detailed view of supply chain processes and metrics. Metamodel database contains standard SCOR metrics but also enables defining of custom metrics, as well as metrics at lower levels (i.e., Level 4, workflows, or Level 5, transactions).

The developed SCM metamodel enables flexible modelling and creation of different supply chain configurations (models). These models are the basis for the construction of data warehouse (DW) metadata (measures, dimensions, hierarchies, and KPIs).

### 3.2. DW and OLAP KPI Modeling

A user who wants to retrieve information directly from a data source, such as an ERP database, faces several significant challenges.

- The contents of such data sources are frequently very hard to understand, being designed with systems and developers instead of users in mind.
- Information of interest to the user is typically distributed among multiple heterogeneous data sources.
- Whereas many data sources are oriented toward holding large quantities of transaction level detail, frequently the queries that support business decision making involve summary and aggregated information.
- Business rules are generally not encapsulated in the data sources. Users are left to make their own interpretation of the data.

In order to overcome these problems, we have constructed the unified dimensional model (UDM) [44]. The role of a UDM is to provide a bridge between the user and the data sources. A UDM is constructed over one or more physical data sources. The user issues queries against the UDM using a variety of client tools.

Construction of the UDM as an additional layer over the data sources offers clearer data model, isolation from the heterogeneous data platforms and formats, and an improved performance for aggregated queries. UDM also allows business rules to be embedded in the model. Another advantage of this approach is that UDM does not require data warehouse or data mart. It is possible to construct UDM directly on top of OLTP (on-line transactional processing) systems and to combine OLTP and DW systems within a single UDM.

In the UDM it is possible to define cubes, measures, dimensions, hierarchies, and other OLAP elements, from the DW schemas or directly from the relational database. This enables providing the BI information to the business users even without previously built DW, which can be very useful having in mind the fact that within the supply chain there can be many nonintegrated data sources which require time to connect, integrate, and design the data warehouse.

Flexibility of UDM is also manifested in the fact that tables and fields can be given names and descriptions that are understandable to the end-user and hide unnecessary system fields. This metadata is further used throughout the UDM, so all the measures, dimensions, and hierarchies that are created based on these table fields will use these new names.

Definitions of all UDM elements are stored as XML (eXtensible markup language) files. Each data source, view, dimension, or cube definition is stored in a separate XML file. For dimensions, these files contain data about tables and fields which store dimension members. OLAP cube definition files also contain information on how the preprocessed aggregates will be managed. This metadata approach enables centralized management of the dimensional model for the entire supply chain and provides an option for model integration and metadata exchange.

Measures are one of the basic UDM elements. Measures are the primary information that business users require in order to make good decisions. Some of the measures that can be used for the global supply chain analysis and monitoring are as follows [21]:

- reliability:
  1. perfect order fulfillment,
- responsiveness:
  1. order fulfillment cycle time,
- agility:
  1. upside supply chain flexibility,
  2. upside supply chain adaptability,
  3. downside supply chain adaptability,
  4. overall value at risk,
- cost:
  1. total cost to serve,
- asset management efficiency:
  1. cash-to-cash cycle time,
  2. return on supply chain fixed assets,
  3. return on working capital.

Each of these measures is calculated by using the lower-level metrics from all supply chain participants. For example, the perfect order fulfillment measure is based on the performance of each level 2 component of the order line to be calculated (% of orders delivered in full, delivery performance to customer committing date, documentation accuracy, and perfect condition).

During the design, for each measure we need to define the following properties:

- name of the measure,
- what OLTP field or fields should be used to supply the data,
- data type (money, integer, or decimal),
- formula used to calculate the measure (if there is one).

The next step is to cluster measures into measure groups. The measure groups are an integral part of the UDM and the cube. Each measure group in a cube corresponds to a table in the data source view. This table is the measure group's source for its measure data.

Supply chain process model (built using the SCM metamodel) can be used as the basis for defining measures and measure groups because it provides relationships between business processes and metrics, metrics hierarchies, definitions, quantitative and qualitative descriptions, and description of possible data sources that provide data for calculations.

Companies often define key performance indicators, which are important metrics used to measure the health of the business. An OLAP KPI is a server-side calculation meant to define company's most important metrics. These metrics, such as net profit, assets utilization, or inventory turnover, are frequently used in dashboards or other reporting tools for distribution at all levels throughout the supply chain.

The UDM allows such KPIs to be defined, enabling a much more understandable grouping and presentation of data. Key performance indicator is a collection of calculations that are associated with a measure group in a cube that is used to evaluate business success. Typically, these calculations are a combination of multidimensional expressions (MDX) or calculated members. KPIs also have additional metadata that provides information about how client applications should display the results of the KPI's calculations. The use of OLAP-based KPIs allows client tools to present related measures in a way that is much more readily understood by the user.

Table 1 lists common KPI elements and their definitions [39].

**Figure 4:**
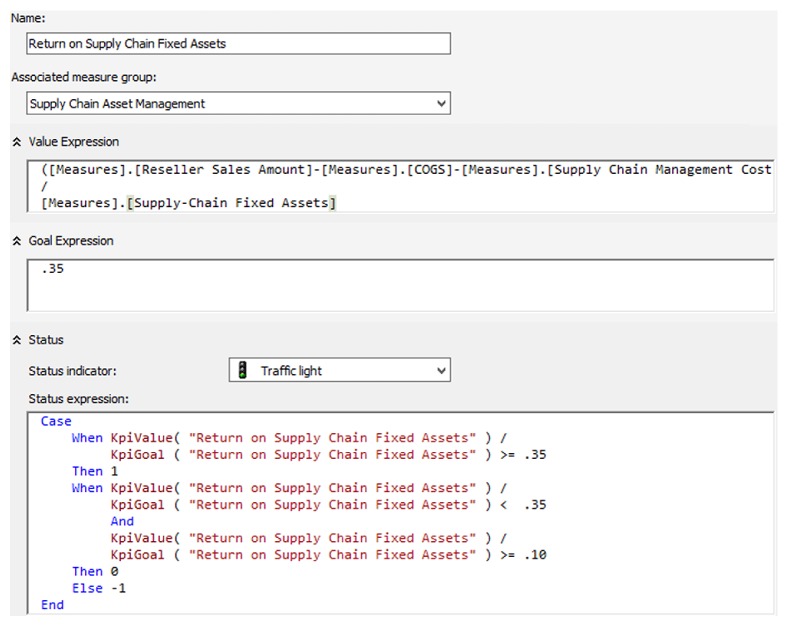
DX KPI definition.

Figure 4 shows a part of the return on supply chain fixed assets KPI defined in the OLAP server.

Besides aforementioned benefits of the UDM model, it also serves as a basis for data mining model design because it provides a consolidated, integrated, aggregated, and preprocessed data store.

## 4. Data Mining Model for KPI Prediction

While proliferation of reporting and multidimensional analytics has greatly benefited many organizations of all sizes, the next step in promoting business agility and operational efficiency is to make the leap from retrospective analysis of historical data to proactive actions based on predictive analysis of business data and to embed intelligent, fact-based decision making into business processes. The key to accomplishing this is to use powerful data mining algorithms to analyze large data sets, compare new data to historical facts and behaviors, identify classifications and relationships between business entities and attributes, and deliver accurate predictive insights into all of the systems and users who make business decisions.

Building a data mining model is a part of a larger process that includes everything from defining the basic problem the model will solve to deploying it into a working environment. A model typically contains input columns, an identifying column, and a predictable column. Data type for the columns can be defined in a mining structure based on which algorithms process the data. Depending on the case, a column can be the following:

- continuous column: this column contains numeric measurements typically the product cost, salary, account balance, shipping date, and invoice date having no upper bound.
- discrete column: these are finite unrelated values such as product category, location, age, and telephone area codes. They do not need to be numeric in nature and typically do not have a fractional component.
- discretized column: this is a continuous column converted to be discrete, for example, grouping salaries into predefined bands.
- key: the column which uniquely identifies the row, similar to the primary key.

Different models for a given business problem could be used for analyzing various business scenarios, identifying the analytical requirements, tuning the parameters, and evaluating the results of the models to make a business decision.

Predictive models can be used to forecast explicit values, based on patterns determined from known results. For example, we can define the target customer service level KPI and set the target to 95%. Then, based on the historical data, a model can be built that predicts this KPI in the future.

There is a variety of techniques developed to achieve that goal—typically applying different models to the same data set and then comparing their performance to choose the best one. For the KPI prediction, different DM models and algorithm can be used depending on the goal and business case. Here, the details about considering various models and choosing the best one based on their predictive performance (i.e., explaining the variability in question and producing stable results across samples) are briefly explained.

- Classification algorithms (such as decision trees) predict one or more discrete variables, based on the other attributes in the dataset.
- Regression algorithms predict one or more continuous variables, such as profit or loss, based on other attributes in the dataset.
- Time series algorithms forecast the patterns based on the current set of continuous predictable attributes.
- Association algorithms find correlations between different attributes in a dataset. The most common application of this kind of algorithm is for creating association rules, which can be used in a market basket analysis or KPI analysis.

Choosing the right algorithm to use for a specific business task can be challenging. While it is possible to use different algorithms to perform the same business task, each algorithm produces a different result, and some algorithms can produce more than one type of result. For example, decision trees algorithm can be used not only for predictions, but also as a way to reduce the number of columns in a dataset, because the decision tree can identify columns that do not affect the final mining model. The type of algorithm depends on the type of prediction. For example, if we are predicting a discrete attribute (i.e., out-of-stock) we can use naïve Bayes, decision trees, or neural networks. If we are predicting a continuous attribute (i.e., supply chain sales amount), a time series algorithm can be used. If we are predicting a sequence (i.e., analyzing the factors leading to delivery failure), a sequence clustering can be used. Using more than one data mining model over the same mining structure is a good practice, since the best model can be selected for predictions. Lift charts can be useful tool to check the accuracy of the data mining models once built on the input data.

In contrast to standard KPIs that only report past or at best the present performance, predictive KPI looks forward and use data mining to show what the situation will be in the next month, quarter, or year. For example, we can use customer data to predict a future demand and thus better plan the production and inventory management processes. Or we can predict disruption in delivery and proactively plan alternative delivery modes.

This allows organization to react before certain disruption happens. Predictive KPI can give the insight into emerging trends or into potential opportunities or problems.

The advantage of using the OLAP-based KPIs is that they are server based and can be consumed by a variety of clients. This means that each client throughout the supply chain will access a single version of the truth, thus making coordination efforts much easier. Also, making a complex calculation on the server can have performance benefits.

### 4.1. Building Prediction Data Mining Models

In this section, two approaches for building KPI prediction models within the UDM are introduced:

- using OLAP data mining dimensions,
- using prediction tables.

Data mining dimensions are results of predictive calculations which are saved into the cube as new dimensions. These dimensions can be browsed or even sliced and diced by results of predictions just as with any other dimension. The special MDX* predict* function can be used to perform predictions. This allows performing prediction joins against data mining models from queries within the cube. When calculating KPI elements such as value, target, or status, we make use of the data mining dimension.

This procedure can be used for various supply chain analysis tasks such as inventory out-of-stock prediction, supplier lead time prediction, and forecasting of customer demand or order fulfillment time. For these tasks, different data mining algorithms such as decision trees, time series, or neural networks can be used. Models can be evaluated and compared in terms of accuracy and precision.

The alternative way for making KPI predictions is using the prediction tables. Prediction tables are just any other tables used for an OLAP cube. They can be a measure group or a dimension, but typically they will be a measure group. In this approach data mining predictions are performed within the ETL (extract, transform, and load) process. ETL package pulls data from the data source, performs a prediction task, and loads the results into a new prediction table. This gives us greater flexibility because we can add a new table to the data warehouse. It is also more flexible for scheduling the training of the model and for maintaining the model. The model can be defined outside the cube and does not need to be processed along with the cube. So, the model can be replaced if we find the better model without altering the cube.

**Figure 5:**
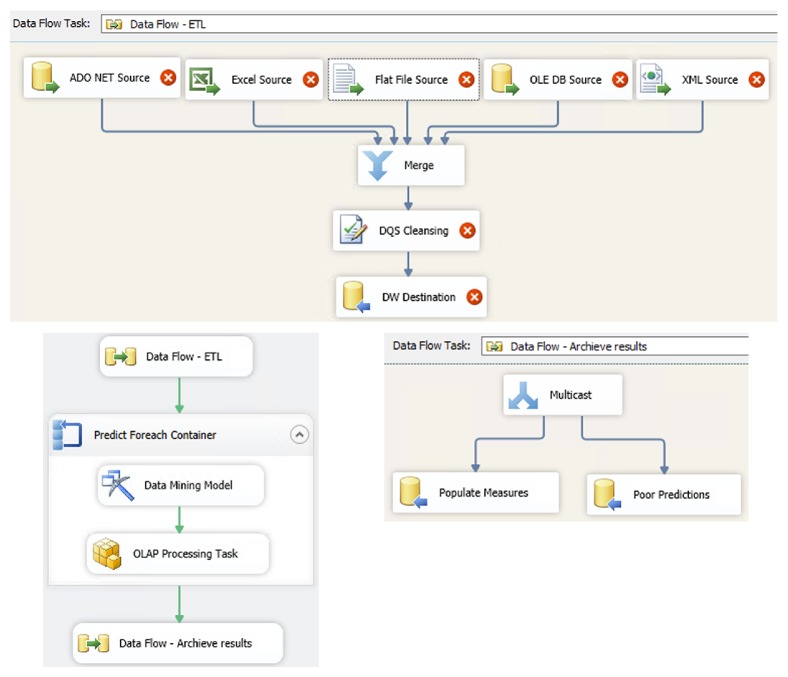
Data mining ETL package.

Figure 5 shows an example of the ETL data and control flows that perform data extraction, integration, cleansing, and data mining prediction.

In the first step ETL package pulls data from different supply chain sources and passes it to a certain data processing component and then into the prediction component and data mining query. This can call any OLAP server or data mining model and return the results in the ETL pipeline. Then, we can perform different operations: populate measures, split predictions into good and poor predictions, or define any type of filtering or modifications. In order to design such ETL packages we used special ETL components such as a data mining model training destination for training data mining models and a data mining query transformation that can be used to perform predictive analysis on data as it is passed through the data flow. The MDX expressions for building KPIs over prediction tables are just the same as for any other KPIs.

Both presented predictive modeling approaches can be used for making the KPI predictions, so the designers have possibility chosen appropriate design approach. The selection depends on the particular scenario. Generally, data mining dimensions offer slightly better performance and slice and dice capabilities, but data mining models must be within the same cube which means it is not possible to use data mining models from another cube or server. On the other hand, the approach with prediction tables performs predictions within the ETL service, instead of the OLAP service. This imposes some additional load on the server during ETL package execution, whereas OLAP cubes can be preprocessed before deployment. However, prediction tables offer more flexibility in terms of scheduling and maintenance, and the models can be defined and maintained outside the cube or replaced with better (more accurate) models without altering the cube. Also, this approach is more suitable for integration scenarios, where supply chain partners can have different analytical systems.

### 4.2. Validation of Data Mining Prediction Models

Before deploying and utilizing prediction models into production, they must be validated. This is a very important step in the data mining process because we need to know how well models perform against actual data. For the validation of the proposed predictive models, a real-world data set from the automotive company was used.

There is no single all-inclusive method which can prove quality of the data and the model. There are several approaches for evaluating the quality of a data mining prediction model. We can use various statistical techniques or involve supply chain domain experts to analyze the prediction results. Furthermore, we can split existing data set into training and testing sets in order to check the accuracy of the model. The training set is used to create the mining model. The testing set is used to test model accuracy.

**Figure 6:**
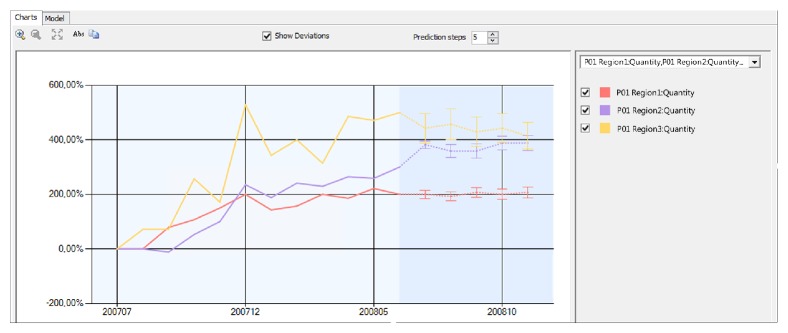
Forecasting data with deviations.

These approaches are not mutually exclusive but can be combined together during design and testing phases to refine models through series of iterations. Various tools can be used for testing data mining prediction model: lift charts, profit charts, scatter plots, classification matrix, cross-validation, and so forth.  Figure 6 illustrates sales quantity trends and predictions deviation for a single product at three different geographic regions.

Validation needs to include different measures which relates to accuracy, reliability, and usefulness of the prediction models. Accuracy tells us how well the model correlates the results with the attributes in the data set. Reliability is also very important characteristic of the prediction models which shows how effective the model is with different data sets. This is especially significant in supply chains that include different divisions and organizations with various data sets. If the model produces similar types of predictions or kinds of patterns, it can be considered reliable.

And finally, data mining models have to be useful, meaning that they need to provide certain answers and to support the decision-making process. For example, if percentage of orders that are fulfilled on the customer's originally committed date is decreasing, we need to know why. This is where the key influence analysis comes into action.

### 4.3. End-User Analytics

Once we define OLAP-based predictive KPIs, we can use different client applications for browsing and for slicing and dicing based on various criteria. For example, UDM model enables slicing data by different dimensions (i.e., organization, geography, product, etc.) or dimension hierarchies. Furthermore, data can be filtered by particular values. For example, we can display prediction of the cash-to-cash cycle time KPI for particular year and quarter, country, and organization.

Additionally, predictive analysis can detect attributes that influence KPIs. Business users can monitor trends and analyze key influencers in order to identify those KPIs (attributes) that have a sustained effect or significant positive or negative impact, for example, identifying whether price discount on a certain product has long-term impact on sales or only produces a short-term effect. Such actionable insights enable companies to better plan improvement strategy and improve their responsiveness.

The UDM also allows the option to define actions in relation to query results. It provides a way to define actions that a client can perform for a given context. This feature goes further than traditional analytical applications which only present data. Furthermore, it provides mechanism to discover problems and deficiencies, thus improving the supply chain performance. An action can start a specific application or load information from a database or a data warehouse. For example, a drill-through action can show detailed rows behind a total, or a reporting action can launch a report based on a dimension attribute's value (parameters can be passed via URL). Hyperlink actions can open specific pages or applications such as a web page showing SCOR recommended best practices for particular process. Actions can be specific to any displayed data, including individual cells, dimension members, or KPIs, resulting in more detailed analysis or even integration of the analysis application into a larger data management framework.

After using the data mining for predictive KPIs, it is possible to use different client applications and technologies such as web portal dashboards, scorecard systems, spreadsheets, web services, or feeds to display and analyze information.

## 5. Supply Chain Intelligence Web Portal for Predictive Analytics

A predictive analysis solution is most effective when it is pervasive throughout the organization and helps to drive day-to-day decisions across the business with its scale and enterprise-level performance. Furthermore, providing a way to implement comprehensive predictive analysis intuitively enables self-service data mining for users, which in turn enables the business to promptly gain actionable insights.

In order to overcome the shortcomings of the existing BI and PM client tools, the specialized web portal that enables supply chain users to monitor business processes, collaborate, and take actions is designed [44]. It has been successfully implemented as a pilot project in an automotive company. Automotive supply chain is typically very complex, with many organizations, intertwined processes, and multitude of users with different requirements.

The portal represents a single point of access to all relevant information in a personalized and secured manner. Its composite and service-oriented architecture enables inclusion of different PM components and tools (KPIs, dashboards, scorecards, strategy maps, reports, etc.). PM elements can be personalized and adjusted, and information can be filtered just by using a web browser. PM elements can be defined within the portal and also embedded from the external source (OLAP, another application, or spreadsheets) via web services. This information is presented through special analytical web parts. The portal itself can be a provider (via web services or RSS-really simple syndication) to other applications.

All these capabilities make the proposed PM model and the software system extremely flexible and applicable in various supply chain scenarios and different industries. Because they are based on the SCM metamodel and use a standardized metrics they can be used in various business domains. On the other hand, software system architecture enables relatively easy and fast customization and extensibility. For example, existing KPI components can be easily reused many times, and only needed items can be added to web sites and pages. Also, new (custom) lower-level metrics can be defined in the SCM metamodel and realized in the data warehouse, thus allowing company-specific and industry-specific performance measurement.

With PM portal capabilities, supply chain partners and teams can do the following:

- use a predefined PM portal template with out-of-the-box modules optimized for access and management of reports, data connections, spreadsheets, and dashboards. Dashboard pages can contain several web parts, each of them showing information from different data sources.
- communicate strategy and monitor its execution at different levels of the supply chain. KPIs status and trend can be tracked using the special KPI and scorecard web parts. KPIs can display information from different data sources (e.g., OLAP cubes or spreadsheets). The portal also supports the concept of strategy maps by providing a specialized module providing a hierarchical view of the KPI measures across levels of the organization by presenting relationships, priorities, and perspectives [44]. Strategy map can be generated automatically, based on a particular scorecard. Each element on the map is highlighted with appropriate color. This enables visual performance tracking in relation to predefined strategy.
- customize and personalize sites, pages, or modules by adding or removing certain web parts and by applying filter web parts. Filters allow dashboards to be personalized by communicating shared parameters amongst web parts on a dashboard. For example, the current user filter web part automatically filters information based on who is logged on to the computer. This is useful for display of personal information such as customer accounts or tasks that is currently assigned to that user.

**Figure 7:**
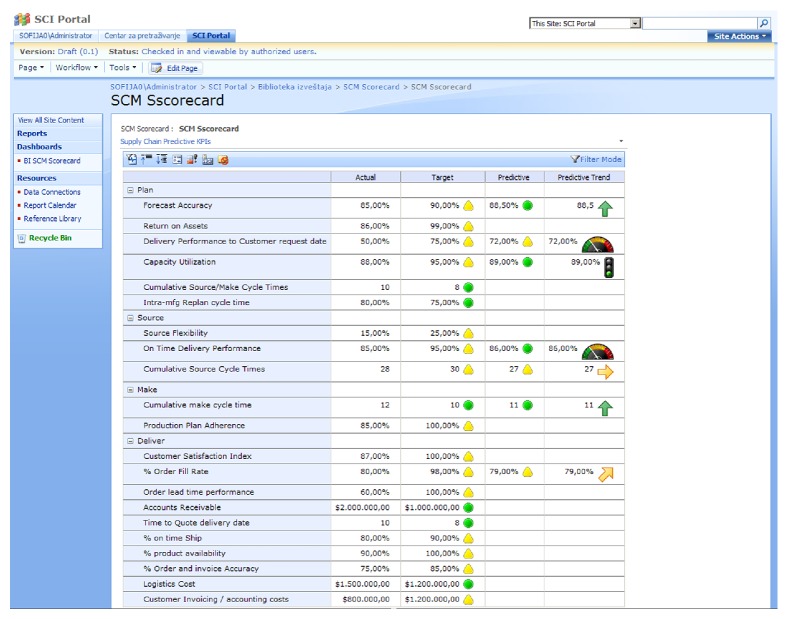
Supply chain scorecard with predictive KPI.

Figure 7 shows a specialized SCM scorecard for global supply chain performance management.

It is constructed based on top of the OLAP KPIs, which are again based on the SCM process model and metrics. KPI are created by SCM segments (plan, source, make, deliver, and return) as hierarchies, so it is possible to perform drill-down analysis, track performance against defined goals, and get future performance values and trends.

The presented solution is very flexible in terms of presenting the KPIs. Owing to several specific BI web parts, the portal can display KPIs from the OLAP server, spreadsheets, and other sources (portals, report servers, etc.).

The dashboard page can display numerous metrics and views business on a single screen. The portal supports quick deployment of dashboards assembled from web parts. Each web part can contain a particular view or metric, and users can customize their individual dashboards to display the views that are most meaningful to them, such as those with the metrics they need to monitor on a daily basis.

Additionally, portal supports events and automatic alerting. Users can subscribe to specific documents or keywords and categories, to be notified (via email, SMS, or web feed) when metrics are updated or new intelligence becomes available. They can also use other features, such as planning, enterprise search, subscription, and routing functions, to work with team members on a single item (i.e., scorecard, KPI, etc.) and to automate collaborative performance management processes. The portal also provides fine-grained authentication and authorization which enable secure access and content personalization.

PM web portal enables business users to define and use scorecards and key performance indicators to drive accountability and alignment across the supply chain. Scorecards and KPIs reflect planning, budgeting, and forecasting changes in real time to help users understand the business drivers, challenges, and opportunities they face. Monitoring becomes a part of the regular, day-to-day management process.

The proposed PM model and the software system are successfully used in the automotive company for modeling and design of the supply chain PM system. The system was used by many different users (low-level, midlevel, and top-level managers) for monitoring performance and making KPI predictions and collaborative decision making. The users reported a high level of satisfaction highlighting the following features and benefits: global performance view, standardized metrics, proactive management through predictions, high data quality, on-demand information and services, easy to use and intuitive web-based interface, personalization, customization, and collaboration services.

## 6. Conclusions

The goal of supply chain performance management is to help decision makers better manage, plan, understand, and leverage their performance. Performance management includes monitoring, measurement, and analysis of various performance data and also collaborative decision making and synchronization.

Performance management is critical to the ultimate success of complex business systems such as supply chains. Key performance indicators are used to measure supply chain performance on a strategic, tactical, and operational level. Unfortunately, most of the existing KPI systems are backward looking, isolated, and static. Also, they lack the ability to efficiently deliver information to decision makers.

In the fast-changing and volatile business environment where companies are competing as part of supply chains, it is no longer sufficient to react to problems after they occur, but to anticipate future performance and intelligently recommend appropriate actions.

Predictive analytics is a natural complement to traditional PM software and processes. While most of existing supply chain PM systems present information about what has happened, predictive PM systems can provide information about what will happen and also why something happened and what should be done to resolve performance problems.

The presented supply chain PM model takes a unified approach to performance management with all the elements required for the next generation of PM systems. The main benefits of this approach and PM software solution can be summarized as follows.

- Extracting additional value from existing data repositories: supply chain information systems hold a large volume of data. With predictive analytics, a new knowledge can be extracted, thus providing better projections about future performance.
- Global approach to supply chain performance management: process model and metrics enables standardized performance measurement across all levels in supply chain hierarchy. Approach with data warehouse provides cleaned and consolidated data repository which can be used for data mining predictions.
- Knowledge-based planning and strategy development: BI tools and technologies such as data mining and multidimensional analysis enable better management through more informed decision making. This provides enhanced scenario and risk analysis, improved planning, and ultimately development of optimal supply chain strategies.
- Transition from reactive actions to proactive programs: employing predictive data mining models inside decision-making processes allows supply chain members to react timely and to better adapt to changes.
- Achieving a competitive advantage application of predictive analytics can enable a competitive advantage through better adaptivity, less risk, and improved responsiveness.
- Collaborative and pervasive intelligence and performance monitoring: PM web portal provides a complete, intuitive, and collaborative business ecosystem that extends the insight of predictive analysis to inform business decisions throughout the supply chain.

This makes the presented supply chain PM model and software solution an excellent environment to create applications that contain key features of future PM systems like visual intelligence, collective intelligence, predictive analytics, and real-time insight delivery.

## Figures and Tables

**Table 1:** OLAP KPI structure.

Term	Definition
Goal	An MDX numeric expression that returns the target value of the KPI.
Value	An MDX numeric expression that returns the actual value of the KPI.
Status	An MDX expression that represents the state of the KPI at a specified point in time. The status MDX expression should return a normalized value between −1 and 1.
Trend	An MDX expression that evaluates the value of the KPI over time. The trend can be any time-based criterion that makes sense in a specific business context.
Status indicator	A visual element that provides a quick indication of the status for a KPI. The display of the element is determined by the value of the status MDX expression.
Trend indicator	A visual element that provides a quick indication of the trend for a KPI. The display of the element is determined by the value of the trend MDX expression.
Display folder	The folder in which the KPI will appear to the user when browsing the cube.
Parent KPI	A reference to an existing KPI that uses the value of the child KPI as part of the KPI's computation.
Current time member	An MDX expression that returns the member that identifies the temporal context of the KPI.
Weight	An MDX numeric expression that assigns a relative importance to a KPI. If the KPI is assigned to a parent KPI, the weight is used to proportionally adjust the results of the KPI value when calculating the value of the parent KPI.

## References

[1] Handfield B. R., Nichols L. E. (2002). *Supply Chain Redesign: Transforming Supply Chains into Integrated Value Systems*.

[2] Chan F. T. S., Chan H. K., Qi H. J. (2006). A review of performance measurement systems for supply chain management. *International Journal of Business Performance Management*.

[3] Gunasekaran A., Patel C., Tirtiroglu E. (2001). Performance measures and metrics in a supply chain environment. *International Journal of Operations and Production Management*.

[4] Vesset D. (2007). *Worldwide Business Analytics Software 2007–2011 Forecast: The Growth Cycle Continues*.

[5] Lapide L. (1999). What about measuring supply chain performance?. *Achieving Supply Chain Excellence through Technology*.

[6] Microsoft (2008). *Predictive Analysis with SQL Server*.

[7] Shepherd C., Günter H. (2006). Measuring supply chain performance: current research and future directions. *International Journal of Productivity and Performance Management*.

[8] Estampe D., Lamouri S., Paris J.-L., Brahim-Djelloul S. (2013). A framework for analysing supply chain performance evaluation models. *International Journal of Production Economics*.

[9] Arzu Akyuz G., Erman Erkan T. (2010). Supply chain performance measurement: a literature review. *International Journal of Production Research*.

[10] Gopal P. R. C., Thakkar J. (2012). A review on supply chain performance measures and metrics: 2000-2011. *International Journal of Productivity and Performance Management*.

[11] Lappide L. (2010). Predictive metrics. *The Journal of Business Forecasting*.

[12] Bauer K. Predictiveanalytics: the next wave in KPIs. http://www.information-management.com/issues/20051101/1040476-1.html.

[13] Beamon B. M. (1999). Measuring supply chain performance. *International Journal of Operations and Production Management*.

[14] Lambert D., Pohlen T. (2001). Supply chain metrics. *International Journal of Logistics Management*.

[15] Cai J., Liu X., Xiao Z., Liu J. (2009). Improving supply chain performance management: a systematic approach to analyzing iterative KPI accomplishment. *Decision Support Systems*.

[16] Gunasekaran A., Patel C., McGaughey R. E. (2004). A framework for supply chain performance measurement. *International Journal of Production Economics*.

[17] Kaplan R., Norton D. (2006). *Strategy-Focused Organization: How Balanced Scorecard Companies Thrive in the New Business Environment*.

[18] Bhagwat R., Sharma M. K. (2007). Performance measurement of supply chain management: a balanced scorecard approach. *Computers and Industrial Engineering*.

[19] Stefanovic N., Stefanovic D., Bramer M. (2009). Supply chain business intelligence: technologies, issues and trends. *Artificial Intelligence: An International Perspective*.

[20] McDonald K., Wilmsmeier A., Dixon C. D., Inmon W. H. (2006). *Mastering the SAP Business Information Warehouse*.

[21] https://supply-chain.org/f/SCOR11QRG.pdf.

[22] Kocaoğlu B., Gülsün B., Tanyaş M. (2013). A SCOR based approach for measuring a benchmarkable supply chain performance. *Journal of Intelligent Manufacturing*.

[23] Shapiro F. J. (2001). *Modeling the Supply Chain*.

[24] Chae B. K., Olson D. L. (2013). Business analytics for supply chain: a dynamic-capabilities framework. *International Journal of Information Technology & Decision Making*.

[25] Raisinghani S. M., Singh K. M., Wang J. (2008). Data mining for supply chain management in complex networks. *Data Warehousing and Mining: Concepts, Methodologies, Tools, and Applications*.

[26] Dignan L. Data depot. http://www.baselinemag.com/c/a/Projects-Supply-Chain/Data-Depot.

[27] Hofmann M. (2004). *Best Practices: VW Revs Its B2B Engine*.

[28] Dowse S. http://www.predictiveanalyticsworld.com/patimes/why-supply-chains-need-business-intelligence.

[29] Mathieu R., Levary R. R., Wang J. (2009). Data warehousing and mining in supply Chains. *Encyclopedia of Data Warehousing and Mining*.

[30] Predictive inventory management: Keeping your supply chain in balance. ftp://ftp.boulder.ibm.com/common/ssi/ecm/en/ytw03260usen/YTW03260USEN.PDF.

[31] Dhond A., Gupta A., Vadhavkar V. Data mining techniques for optimizing inventories for electronic commerce.

[32] Symeonidis A. L., Nikolaidou V., Mitkas P. A. Exploiting data mining techniques for improving the efficiency of a supply chain management agent.

[33] Stefanovic N., Stefanovic D., Radenkovic B., Ellis R., Allen T., Petridis M. (2008). Application of data mining for supply chain inventory forecasting. *Applications and Innovations in Intelligent Systems XV*.

[34] Gheorghe C. (2006). *Predictive Analytics: BPM Drives the Dynamic Organization*.

[35] Eckerson W. W. (2006). *Performance Dashboards Measuring, Monitoring, and Managing Your Business*.

[36] Seifert M., Eschenbaecher J., Luis C. M. (2005). Predictive performance measurement in virtual organisations. *Emerging Solutions for Future Manufacturing Systems*.

[37] Derrouiche R., Holimchayachotikul P., Leksakul K. Predictive performance model in collaborative supply chain using decision tree and clustering technique.

[38] Maleki M., Cruz-Machado V. (2013). Supply chain performance monitoring using Bayesian network. *International Journal of Business Performance and Supply Chain Modeling*.

[39] Stefanovic N., Stefanovic D., Radenkovic B., Mohhebi S., Mahdavi I., Cho N. (2011). Integrated supply chain intelligence through collaborative planning, analytics and monitoring. *Electronic Supply Network Coordination in Intelligent and Dynamic Environment: Modeling and Implementation*.

[40] Stefanovic D., Stefanovic N., Radenkovic B. (2009). Supply network modelling and simulation methodology. *Simulation Modelling Practice and Theory*.

[41] Abukhousa E., Al-Jaroodi J., Lazarova-Molnar S., Mohamed N. (2014). Simulation and modeling efforts to support decision making in healthcare supply chain management. *The Scientific World Journal*.

[42] Stefanovic N., Stefanovic D. (2013). Integrated and interactive software solution for knowledge-based supply network design. *Computer Systems Science and Engineering*.

[43] Stefanovic D., Stefanovic N. (2008). Methodology for modeling and analysis of supply networks. *Journal of Intelligent Manufacturing*.

[44] Stefanović N., Stefanović D. (2011). Supply chain performance measurement system based on scorecards and web portals. *Computer Science & Information Systems*.

